# Culture-independent genome sequencing of *Coxiella burnetii* from a native heart valve of a Tunisian patient with severe infective endocarditis

**DOI:** 10.1016/j.nmni.2017.09.004

**Published:** 2017-10-10

**Authors:** J. Delaloye, T. Pillonel, M. Smaoui, A. Znazen, L. Abid, G. Greub

**Affiliations:** 1) Institute of Microbiology, University of Lausanne and University Hospital Center, Lausanne, Switzerland; 2) Intensive Care Unit, Department of Intensive Care Medicine, University of Lausanne and University Hospital Center, Lausanne, Switzerland; 3) Laboratory of Microbiology, CHU Habib Bourguiba Sfax, University of Sfax, Tunisia; 4) Department of Cardiology, Hedi Chaker University Hospital, University of Sfax, Sfax, Tunisia

**Keywords:** Cardiac valve, endocarditis, genomics, intracellular bacteria

## Abstract

We report draft genome of a *Coxiella burnetii* strain sequenced from the native valve of a patient presenting with severe endocarditis in Tunisia. The genome could be sequenced without a cellular or axenic culture step. The MST5 strain was demonstrated to be closely related to the published reference genome of *C. burnetii* CbuK_Q154.

## Introduction

Q fever is a zoonotic infection caused by an obligate Gram-negative intracellular bacterium, *Coxiella burnetii,* which is responsible for acute and persistent infections [1], [2]. Although the acute infection is most often benign and self-limited, chronic manifestations may be life-threatening. In the last few years, reports of Q fever have gradually increased, with an estimated incidence of Q fever of 50 cases per 100 000 people in France [1]. Humans are accidental hosts; sheep and goats are the primary reservoir for *C. burnetii.* Q fever remains asymptomatic in up to 60% of patients. Symptomatic acute Q fever is characterized by three main clinical manifestations: flulike syndrome, pneumonia (with lobar clinical pattern) and transaminitis [3]. Patients with previous valvulopathy or vascular disease, immunocompromised hosts and pregnant women are at high risk for the development of complications of Q fever months or years after the acute disease [4]. Endocarditis is the most frequent and severe clinical manifestation of persistent *C. burnetii* infection and will result in death unless appropriately treated. Infection of vascular aneurysms, cases of chronic hepatitis, osteomyelitis and arthritis are also commonly reported [5], [6].

Although prevalence of anti–*C. burnetii* antibodies appears to be high among blood donors in Tunisia (up to 26%), Q fever infections have been rarely described in Tunisia [7]. A series of 21 cases of acute Q fever have been reported between 2003 and 2007 [8]. Interestingly, hepatitis was the most common clinical presentation. One study of febrile patients identified acute Q fever in 9% of hospital admissions in Tunisia [9]. Only few cases of endocarditis have been described [10], [11], [12]. In a recent systematic review of *C. burnetii* epidemiology in Africa, *C. burnetii* accounted for 1% to 3% of infective endocarditis in Tunisia [13]. More epidemiologic studies are needed to determine the incidence of *C. burnetii* infection in Tunisia, especially in pregnant women.

*C. burnetii* possesses a circular chromosome of about 2 Mbp and a plasmid of 32 to 51 kb, depending on the strain [1]. In recent years, comparative genomic analyses have contributed to the description of the genetic diversity of *C. burnetii* strains around the world and have improved our understanding of the evolution and pathogenesis of *C. burnetii* [14], [15]. Genome sequencing and genetic analyses of *Coxiella*-like tick symbionts support the hypothesis that *C. burnetii* recently evolved from a maternally inherited symbiont of ticks [16]. Gene losses might be implicated in the increased virulence of some strains. Indeed, the loss of the type 1 secretion system (T1SS) in the French Guiana Cb 171 (MST17) epidemic strain might be associated with its increased virulence [17]. Since the first publication of the complete genome sequence of *C. burnetii* in 2003, 11 whole genomes and 27 draft genomes have been released in public databases, including the Dutch veterinary strain NL3262 and its epidemiologically linked human isolate [1], [14], [17], [18], [19], [20]. Nevertheless, the biology of *C. burnetii* is still not fully understood, and comparative genomic analyses might provide useful insights into the pathogenicity of *C. burnetii.*

We report the draft genome sequence of a *C. burnetii* strain directly sequenced from the native valve of a patient presenting with severe endocarditis in Tunisia.

## Clinical case

A 48-year-old man from Sfax, Tunisia, sought care for progressive dyspnoea and leg swelling. He was admitted to a local hospital for chest pain. At admission, the patient was afebrile without haemodynamic instability. Physical examination revealed signs of acute heart failure. Cardiac auscultation revealed a diastolic murmur at the aortic region. Laboratory findings revealed white blood cell count 13 g/L, haemoglobin 84 g/L, platelets 79 000/mm³, C-reactive protein 75.6 mg/L, liver enzyme alteration with aspartate aminotransferase 25 U/L and alanine aminotransferase 171 U/L, and altered kidney function with creatinine 350 μmol/L. Transesophageal echocardiography revealed severe aortic regurgitation, the presence of vegetations on the aortic valve and mild systolic dysfunction. Serial blood cultures remained negative.

A diagnosis of endocarditis was suspected, and the patient was treated empirically with intravenous amoxicillin/clavulanate acid and gentamicin. The patient developed haemodynamic instability and required cardiac surgery with the insertion of a biological prosthesis. The patient experienced postoperative bleeding with disseminated intravascular coagulation and died the day after surgery.

Extended microbiologic investigations were all negative except a serology for *C. burnetii,* which was positive. Patient serum samples displayed strong IgG reactivity (1/6400 for phase I and 1/12 800 for phase II) and a slight IgA antibody reactivity (1/200 for phase I and 1/400 for phase II). No IgM were detected. Culture of the valve remained sterile. Quantitative real-time PCR (qPCR) analysis of the valvular sample was strongly positive (355 395 350 copies/mL) for *C. burnetii.* Whole genome sequencing of the strain was performed directly on the valvular sample to test whether direct sequencing would be feasible for such highly positive sample and to provide genomic data on a Tunisian strain.

## Methods

### Genotyping of strain

Multispacer genotyping (MST) based on the classification scheme of Glazunova *et al.* [21] was performed. Briefly, the sequence of ten intergenic regions of the *C. burnetii* genome (Cox2, Cox5, Cox18, Cox20, Cox22, Cox37, Cox51, Cox56, Co57, Cox61) were used to determined its genotype.

### Whole genome sequencing

Genomic DNA was extracted from the valvular sample with GentleMACS and purified using a Wizard Genomic DNA purification kit (Promega, Madison, WI, USA) and the protocol for animal tissue. Dose before enrichment was 4 ng/μL. Whole genome amplification was performed with the REPLI-g Mini Kit (Qiagen, Germantown, MD, USA) using the protocol for 5 μL of DNA and purified with Agencourt AMPure XP beads (Beckman Coulter, Brea, CA, USA). Bacterial DNA was enriched with NEBNext Microbiome DNA Enrichment Kit (New England Biolabs, Ipswich, MA, USA) and purified with Agencourt AMPure XP beads. The bacterial copy number before and after enrichment was estimated using a specific real-time qPCR targeting the *ompA* gene. Genomic libraries were constructed using the Nextera XT library kit (Illumina, San Diego, CA, USA). Libraries were sequenced on a MiSeq Desktop Sequencer (Illumina). The Illumina 150 bp paired-end reads were filtered and trimmed with fastq-mcf (https://github.com/ExpressionAnalysis/ea-utils), keeping 150 bp reads and an average phred quality score higher than 30 (parameters: ‘-max-ns 1 -l150 -L150 mean-qual- 30’). Filtered reads were mapped to the human genome (accession no. GCA_000001405.23) using BWA mem 0.7.13 [22]. A *de novo* genome assembly of unmapped reads was performed using SPAdes 3.7.1 with kmer lengths ranging from 4 to 127 [23]. Whole genome comparisons were performed with nucmer 3.9.4 from the MUMmer package [24]. A species phylogeny including 11 complete and 28 draft genomes (Supplementary Table S1) was reconstructed with FastTree [25] based on core single-copy orthologs identified with OrthoFinder 0.4.0 [26]. The circular map was drawn with Circos 0.68 [27]. Variants were identified compared to the reference *Coxiella* genome, *Coxiella burnetii* CbuK_Q154 (accession no. GCF_000019885) with snippy 3.7.1 (default parameters, https://github.com/tseemann/snippy). The average nucleotide identity was calculated on the basis of nucmer alignments as described by Richter and Rosselló-Móra [28]. The raw sequencing data and the genome assembly where submitted to the European Nucleotide Archive under the project accession number PRJEB21559.

## Results

**Fig. 1:**
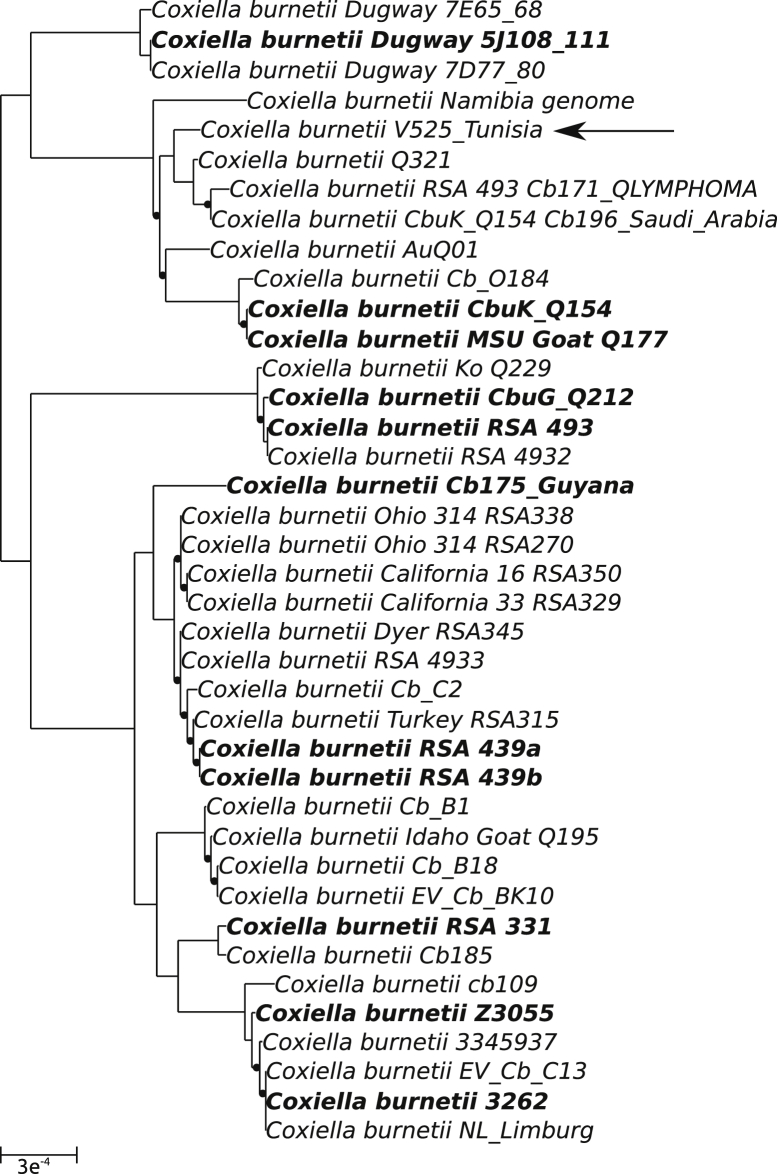
Phylogeny of sequenced *Coxiella burnetii* genomes based on 915 core single-copy orthologs. Eleven complete reference genomes are labelled in bold. Black dots indicate FastTree support values of <1. Black arrow indicates newly sequenced strain V525_Tunisia. Scale indicates number of substitutions per site.

**Fig. 2:**
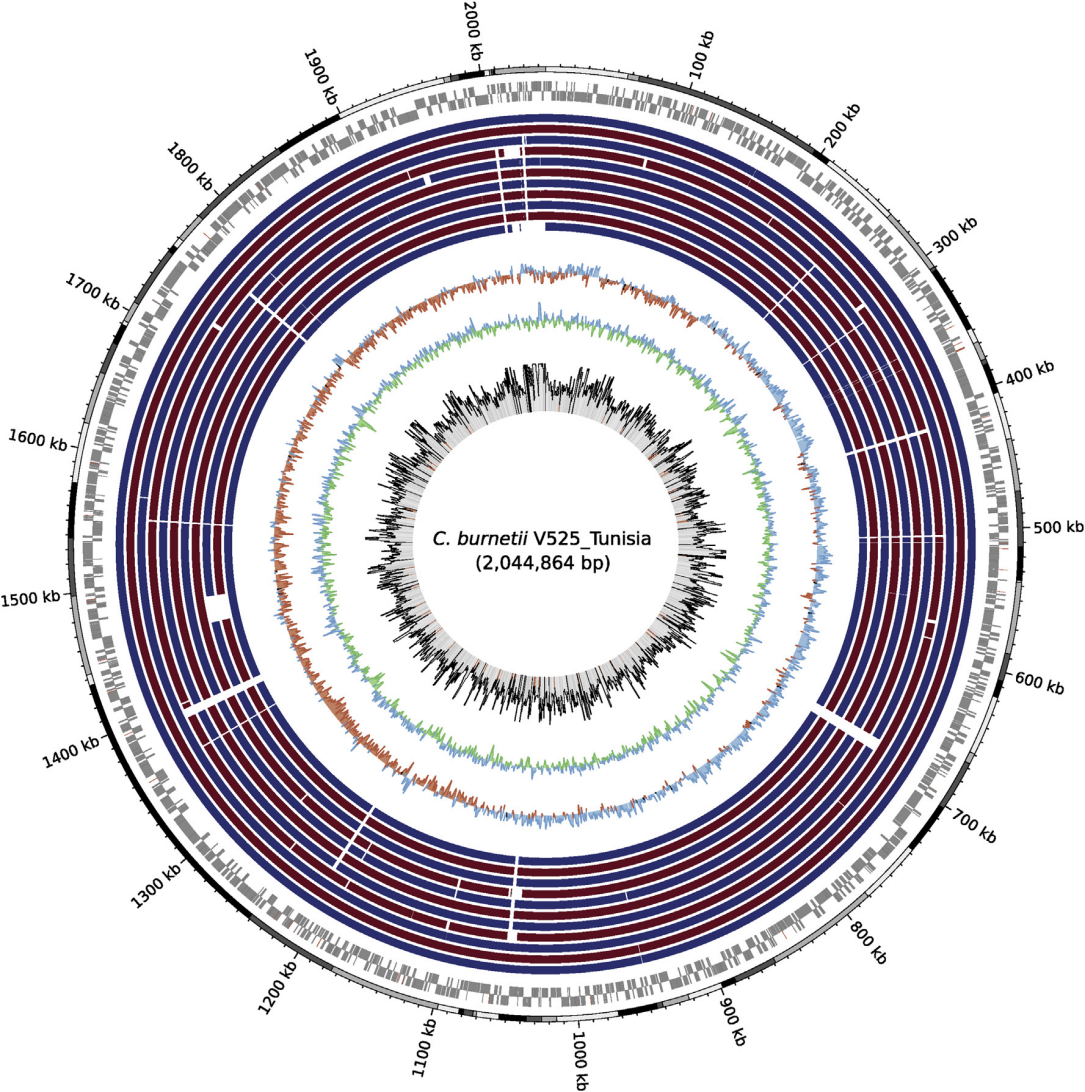
Circular map of 2 044 864 bp assembly. First black and grey outer circle indicates contig boundaries of *Coxiella* draft genome. Contigs were ordered on basis of genome of strain CbuK_Q154. Next two circles report open reading frames of leading and lagging strands. Eleven inner circles report sequence conservation with 11 complete *Coxiella* genomes. From outside to inside: (1) *C. burnetii* CbuK_Q154; (2) *C. burnetii MSU Goat Q177*; (3) *C. burnetii* Dugway 5J108-111; (4) *C. burnetii* CbuG_Q212; (5) *C. burnetii* Cb175_Guyana; (6) *C. burnetii* RSA 493; (y) *C. burnetii* RSA 331; (8, 9, 10) *C. burnetii* strain 3262 (two genomes); (11) *C. burnetii* Z3055. All strains exhibited >99% of average nucleotide identity with reference (Supplementary Table S3). Following two inner circles report GC skew (red for negative, blue for positive) and GC content (blue for above average, green for below average) of reference genome. Innermost circle reports variations in sequencing depth (median depth of 1546×); regions exhibiting sequencing depth of <800× are highlighted in red.

Bacterial DNA enrichment of the clinical sample allowed a sixfold enrichment of *C. burnetii* DNA from 7 241 282 copies/5 μL to 41 632 784 copies/5 μL. Twenty percent of the 12 457 556 good-quality read pairs sequenced from enriched samples were removed after mapping to the human genome. The remaining reads were assembled *de novo.* The median *C. burnetii* sequencing depth was of 1546×. The final assembly resulted in 69 contigs of more than 1000 bp, with a cumulated size of 2 044 864 bp (including the putative plasmid of 34 888 bp) and an N50 of 46 407 bp. The sequenced genome is part of the MST genotype group 5. The phylogeny reconstructed on the basis of the concatenated alignment of 915 single-copy orthologs indicates that this genome is most closely related to published genomes from various parts of the world (Fig. 1), including the United States (strain CbuK_Q154, NC_011528), Australia (strain AuQ01, NZ_JPVV00000000) and Saudi Arabia (Cb196_Saudi_Arabia, NZ_CCXO00000000). *C. burnetii* CbuK_Q154, with an average nucleotide identity of 99.825%, is the closest complete genome currently available (Supplementary Table S2). This genome was used as the reference to identify genetic variants. A total of 2166 variants including 1843 single nucleotide polymorphisms, 131 deletions and 148 insertions were identified (Supplementary Table S3). The genome presented no significant additional content compared to the closely related CbuK_Q154 strain (Fig. 2), and it exhibited no large deletions such as what was observed in the French Guiana Cb 171 (MST17) strain [17].

## Conclusion

We report the draft genome sequence of an MST5 *C. burnetii* strain causing endocarditis in a patient from Tunisia. After a bacterial DNA enrichment step, this genome could be sequenced directly from a cardiac valve tissue without going through a cellular or axenic culture step. Only a few other MST5 strains have been reported so far, and only in France and Tunisia (http://ifr48.timone.univ-mrs.fr/mst/coxiella_burnetii/). More studies are needed to determine which genotypes predominate and if there is a specific clone circulating in Tunisia. The closely related CbuK_Q154 strain was isolated in Oregon, USA, in 1986 from the aortic valve of a person with endocarditis [14]. It is known that *C. burnetii* has a highly clonal population structure [15]. The draft genome sequence of a *C. burnetii* strain causing fulminant endocarditis in Tunisia will help better characterize the genetic diversity and population structure of *C. burnetii* worldwide. Whether given genotypes are associated with some specific clinical presentation and/or severity remains to be determined.

## Conflict of interest

None declared.

## Appendix A Supplementary data

mmc1

mmc2

mmc3
